# Supplementary material for: Deciphering “Immaturity-Stemness” in Human Epidermal Stem Cells at the Levels of Protein-Coding and Non-Coding Genomes: A Prospective Computational Approach
Source: Int J Mol Sci. 2024 Mar 15;25(6):3353. doi: 10.3390/ijms25063353 (PMC10970410; doi:10.3390/ijms25063353)
Supplement: Supplementary file 1 [file ijms-25-03353-s001.zip › ijms-2887060-supplementary.pdf]

International Journal of *Molecular Sciences*

MDPI

Supplementary Materials

Deciphering "Immaturity-Stemness" in Human Epidermal Stem Cells at the Levels of Protein-Coding and Non-Coding Genomes: A Prospective Computational Approach

Tatiana Vinasco-Sandoval [1,2,3,†], Gilles Lemaître [2,4,†], Pascal Soularue [1,2], Michèle T. Martin [1,2] and Nicolas O. Fortunel [1,2,*]

[1] Université Paris-Saclay, 91190 Gif-sur-Yvette, France; gloria.vinasco-sandoval@cea.fr (T.V.-S.); pascal.soularue@cea.fr (P.S.); michele.martin@cea.fr (M.T.M.)
[2] Commissariat à l'Energie Atomique et aux Energies Alternatives (CEA), Laboratoire de Génomique et Radiobiologie de la Kératinopoïèse (LGRK), Institut de Radiobiologie Cellulaire et Moléculaire (iRCM), 2 Rue Gaston Crémieux, 91000 Evry, France; gilles.lemaitre@cea.fr
[3] CEA, Institut de Biologie François Jacob (IBFJ), 92265 Fontenay-aux-Roses, France
[4] Université Paris-Saclay, Univ Evry, 91000 Evry, France
* Correspondence: nicolas.fortunel@cea.fr
† These authors contributed equally to this work.

Supplementary Materials

The following supporting information can be downloaded at: https://www.mdpi.com/article/10.3390/ijms25063353/s1.

Table S1: Sample collection in *KLF4* and *MXD4* studies.
Table S2: Number of enriched terms in individual Over Representation Analysis (ORA).
Table S3: Common differentially expressed lncRNAs identified both in the *KLF4* and in the *MXD4* datasets.
Table S4: ORA selected signature of common differentially expressed protein-coding RNAs identified both in the *KLF4* and in the *MXD4* datasets.
Figure S1: Data preprocessing for the *KLF4* and *MXD4* RNA-seq datasets.
Figure S2: Over representation analysis of common differentially expressed protein-coding RNAs in the *KLF4* and *MXD4* datasets.
Figure S3: Architecture of the computational approach.
Figure S4: Soft thresholding power choice for the *KLF4* and *MXD4* datasets in consensus WGCNA.

Supplementary Tables

Table S1. Sample collection in *KLF4* and *MXD4* studies. Samples corresponding to wild type (WT) and knock-down (KD) cellular contexts.

Model	Sample ID	SRA ID	Cell context
KLF4 (GSE111786)	KLF4_WT1	SRR6829690<br>SRR6829691<br>SRR6829692	WT
	KLF4_WT2	SRR6829693<br>SRR6829694<br>SRR6829695	WT
	KLF4_WT3	SRR6829696<br>SRR6829697	WT
	KLF4_KD1	SRR6829698<br>SRR6829699<br>SRR6829700	KD
	KLF4_KD2	SRR6829701<br>SRR6829702<br>SRR6829703	KD
	KLF4_KD3	SRR6829704<br>SRR6829705<br>SRR6829706	KD
MXD4 (GSE202700)	MXD4_WT1	SRR19165250	WT
	MXD4_WT2	SRR19165251	WT
	MXD4_WT3	SRR19165252	WT
	MXD4_KD1	SRR19165247	KD
	MXD4_KD2	SRR19165248	KD
	MXD4_KD3	SRR19165249	KD

Table S2. Number of enriched terms in individual Over Representation Analysis (ORA). Enriched terms were searched based on 2,311 and 5,200 differential expressed protein-coding RNAs for the *KLF4* and *MXD4* datasets, respectively.

Gene set	Enriched terms*	
	KLF4	***MXD4***
KEGG	27	27
Gene Ontology (GO) – Biological processes	429	353
MSigDB – Hallmarks of genes	12	8
Reactome	27	342
ChEA: ChipX experiment Analysis	37	24
Bioplanet	48	115
Tabula Muris: Single cell transcriptome atlas (*Mus musculus*)	13	7

* Significant enriched terms after hypergeometric test with ORA (FDR < 0.05).

Table S3 and S4 are available as Excel files

Table S3. Common differentially expressed lncRNAs identified both in the *KLF4* and in the *MXD4* datasets.

Table S4. ORA selected signature of common differentially expressed protein-coding RNAs identified both in the *KLF4* and in the *MXD4* datasets.

Supplementary Figures

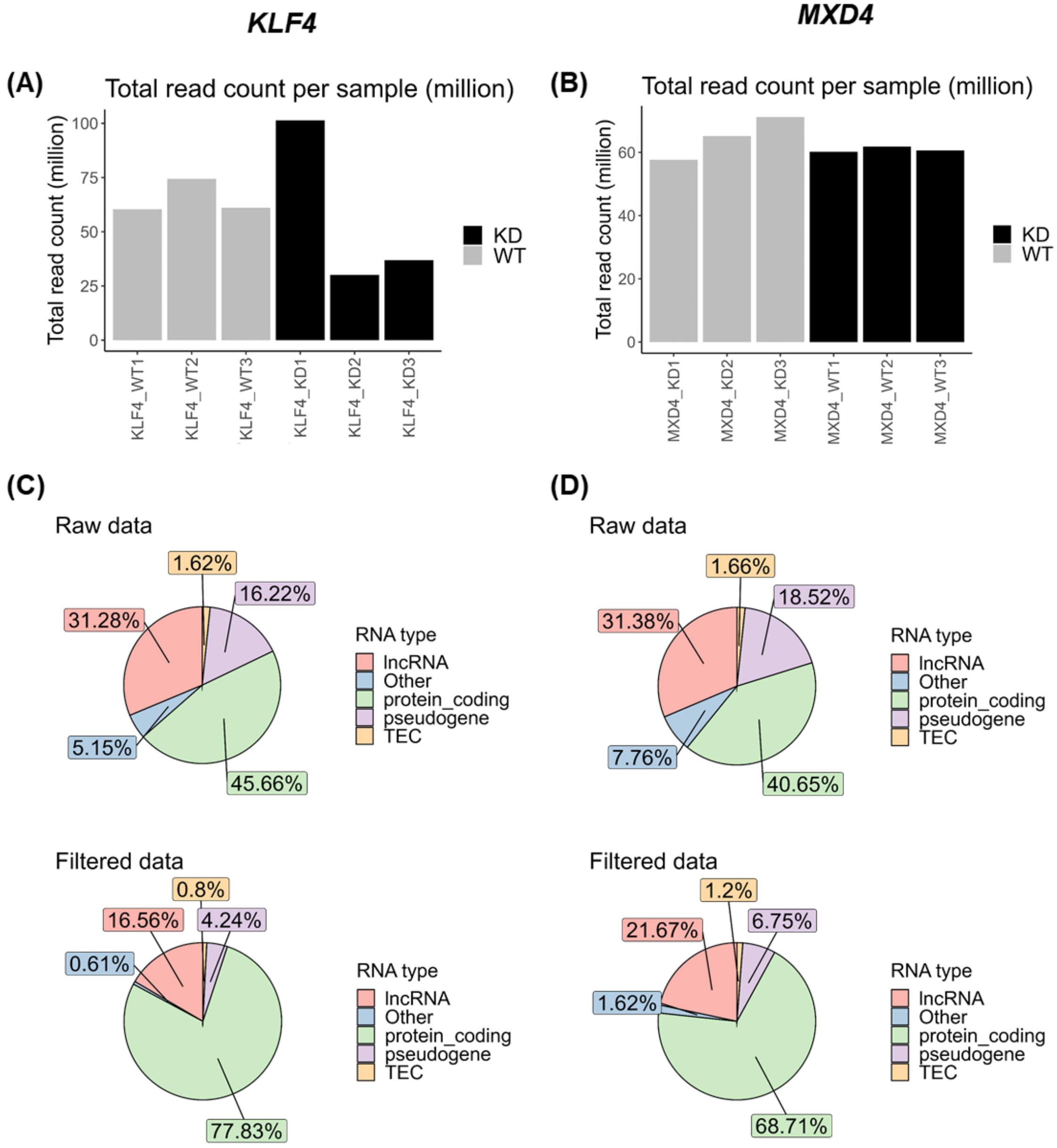

Figure S1. Data preprocessing for the KLF4 and MXD4 RNA-seq datasets. (A-B) Library size in total million read counts per sample for **(A)** *KLF4* and **(B)** *MXD4* datasets. **(C-D)** RNA species distribution before (Raw data) and after filtering low expressed reads (Filtered data) in both datasets,

(C) *KLF4* and **(D)** *MXD4*. Other class includes miRNA, misc_RNA, artifact, IG_genes, Mt_rRNA, Mt_tRNA, snoRNA or rRNA.

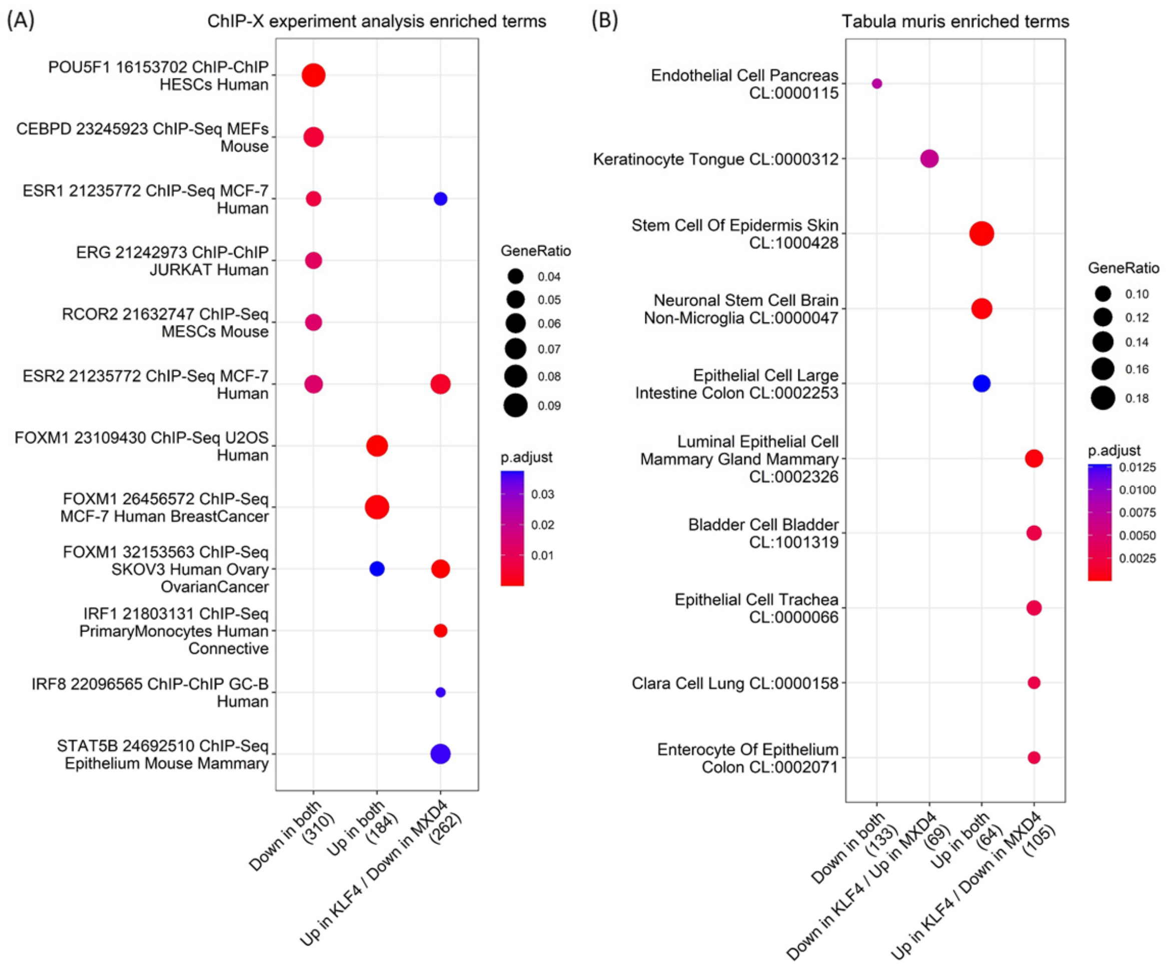

Figure S2. Over representation analysis of common differentially expressed protein-coding RNAs in the KLF4 and MXD4 datasets. Dot plots of enriched terms from (A) ChIP-X experimental analysis and (B) Tabula muris databases. Enrichment was considered significant when False Discovery Rate (FDR) < 0.05.

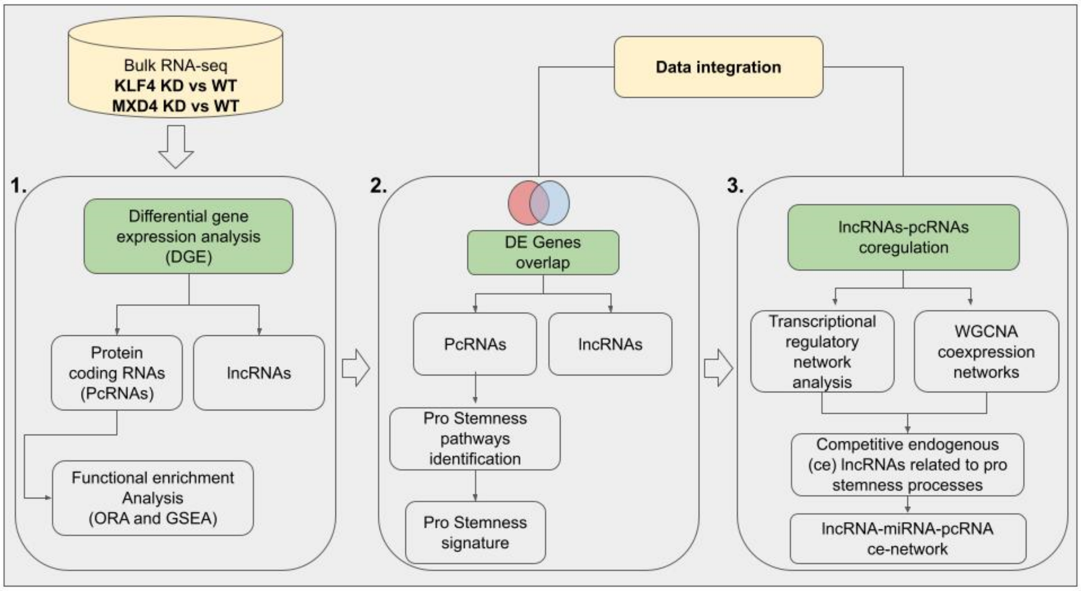

Figure S3. Architecture of the computational approach. Flowchart depicting the main three steps of computational pipeline employed in this study. .

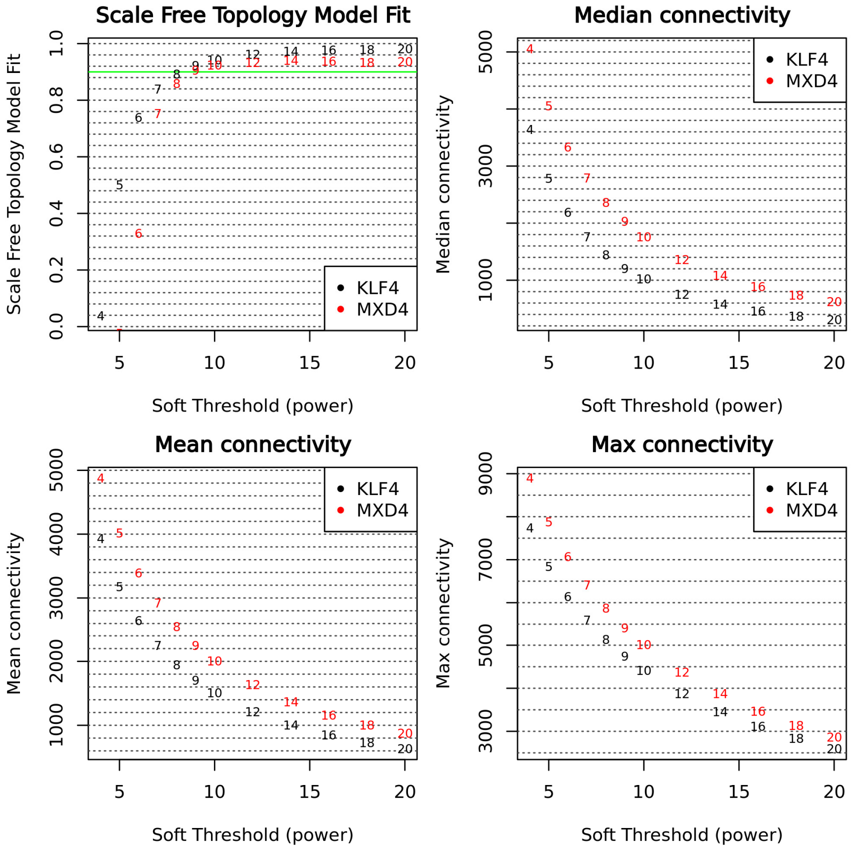

Figure S4. Soft thresholding power choice for the KLF4 and MXD4 datasets in consensus WGCNA. The plots indicate that approximate scale-free topology is attained around the soft-thresholding power of 8 for both models. Numbers inside the plots and x-axes indicate the corresponding soft thresholding powers for network measures (y-axes).
